# Awareness of Breast Cancer Among Male and Female High School Students in Southern Ghana

**DOI:** 10.1155/ijbc/5710341

**Published:** 2025-06-23

**Authors:** Florence Dedey, Josephine Nsaful, Edmund Nartey, Kirstyn E. Brownson, Promise E. Sefogah, Elizabeth Bankah, Theresa Oppong-Mensah, Ernest Amoah Ampah, Mary Efua Commeh, Joe-Nat Clegg-Lamptey

**Affiliations:** ^1^Department of Surgery, University of Ghana Medical School, College of Health Sciences, University of Ghana, Accra, Ghana; ^2^Department of Surgery, Korle Bu Teaching Hospital, Accra, Ghana; ^3^Centre for Tropical Clinical Pharmacology & Therapeutics, University of Ghana Medical School, College of Health Sciences, University of Ghana, Accra, Ghana; ^4^Department of Surgery, University of Utah School of Medicine, Salt Lake City, Utah, USA; ^5^Huntsman Cancer Institute, Salt Lake City, Utah, USA; ^6^Department of Obstetrics and Gynaecology, Korle Bu Teaching Hospital, Accra, Ghana; ^7^Department of Family Medicine, Greater Accra Regional Hospital, Accra, Ghana; ^8^School Health Educational Program, Ghana Education Service, Accra, Ghana; ^9^Non-Communicable Disease Control Program, Ghana Health Service, Accra, Ghana

**Keywords:** breast cancer, breast self-examination (BSE), high school, knowledge, risk factors

## Abstract

**Background:** In Ghana, the incidence of breast cancer is increasing with disproportionately high mortality rates. Awareness about the disease process is critical for achieving early diagnosis of breast cancer in countries without a national screening program. Targeting adolescents in school will help to inculcate good health seeking behaviors with widespread reach. This study assessed the baseline knowledge in high school males and females as an important first step to inform the development of appropriate educational interventions to address identified gaps in student knowledge about breast cancer.

**Methodology:** A multisite cross-sectional study was carried out in 14 high schools in two regions in southern Ghana to assess the baseline student knowledge of breast cancer. Self-administered questionnaires were used covering the following four domains: (1) general breast cancer knowledge, (2) breast cancer symptoms, (3) risk factors for breast cancer, and (4) breast self-examination/screening for breast cancer. For each domain of knowledge tested, the total score was categorized as adequate knowledge (≥ 50% of questions answered correctly) or inadequate knowledge (< 50% of questions answered correctly). Logistic regression was used to determine factors associated with each of the knowledge domains. Stata 14.0 was used for the statistical analysis, and a *p* < 0.05 was considered statistically significant.

**Results:** Nine thousand seven hundred sixty-seven students from 10 coeducational and 4 girls-only schools participated with 68% of respondents being female. The mean student age was 16.9 ± 1.2 years. Eighty-four percent of the students demonstrated adequate general knowledge on breast cancer and 54% demonstrated adequate knowledge of breast cancer symptoms. However, only 34% and 21%, respectively, received a score of adequate knowledge in regard to breast cancer risk factors and BSE/breast cancer screening. After combining all domain scores to evaluate overall breast cancer knowledge, less than half (47%) of the students received an adequate breast cancer knowledge score. Females and the girls-only schools had statistically significant adequate levels of knowledge of breast cancer.

**Conclusion:** The overall knowledge of breast cancer among senior high school students in southern Ghana is inadequate especially on knowledge of breast cancer risk factors, breast self-examination, and breast cancer screening. Breast cancer educational activities should be incorporated into the national school health curriculum in senior high schools across the country to ameliorate this knowledge gap, with special emphasis on risk factors and breast self-examination. Adolescent males should be included in breast cancer education.

## 1. Background

Globally, breast cancer is the most common female cancer with the highest incidence rates in high-income countries (HICs) with an age standardized incidence of 95.1/100,000 [1]. In West Africa, the incidence rates are not as high at 41.6/100,000 but are increasing and predicted to double by 2040 [1, 2]. In addition, mortality rates in low- and middle-income countries (LMICs) are disproportionally high with a mortality to incidence ratio in Western Africa of 0.54 compared to 0.13 in Northern America [1]. Similarly, in Ghana, breast cancer is the most common female cancer and the second most common cause of female cancer-related deaths [1]. Breast cancer is a significant national public health concern given the social and financial impact on individuals diagnosed with this disease [3]. The projected increase in incidence and subsequent mortality can be lessened however [4]. The World Health Organization's (WHO) Global Breast Cancer Initiative is aimed at reducing breast cancer mortality worldwide by 2.5% per year preventing 2.5 million breast cancer deaths by 2040. This initiative is based on three pillars: health promotion for early detection, timely diagnosis, and comprehensive breast cancer management [3]. The aim of this study is to demonstrate baseline knowledge in Ghanaian high school students in order to implement educational initiatives that will promote early detection of breast cancer in the future.

Breast cancer cannot be entirely nor easily prevented, and hence, early detection is crucial in achieving good outcomes [3, 5–7]. Early detection can be achieved through screening to detect asymptomatic cancers or by early diagnosis of symptomatic cancers [3]. Screening has been effective in early detection of breast cancer in HICs with well-established national mammographic screening programs [3]. In Ghana and other LMICs without appropriate infrastructure to support a national mammographic screening program currently, the emphasis is on early diagnosis of symptomatic disease to enable a shift from diagnosis in late to earlier stages [2–4, 8, 9]. Critical to the success of early diagnosis of symptomatic breast cancers is promotion of community fund of knowledge about breast cancer symptoms, risk factors, and methods for early diagnosis as a means to promote earlier recognition of changes in the breast and seeking appropriate healthcare [3, 8, 10–12].

Breast self-examination (BSE) increases breast awareness and can detect symptomatic breast cancers early [13]. Particularly in developing countries with no national mammographic screening, this simple and efficacious practice should be promoted to help in early detection of symptomatic breast cancer [13–15]. Unfortunately, even where awareness of breast cancer may be adequate in developing countries, the practice of BSE is generally low partly due to inadequate knowledge about its practice [13–15].

Although globally most women have heard about breast cancer, breast cancer knowledge and awareness remain low in developing countries [16]. While most breast cancer educational activities target females, educating men about breast cancer could lead to improved support for their partners and female community members in recognizing breast cancer and taking the necessary steps to seek timely treatment [10, 17]. This is of particular importance in Ghanaian society where, culturally, some women depend on their male partner's consent to access healthcare. Noninvolvement of some Ghanaian women in making decisions concerning their health can result in delays in seeking healthcare [18]. Educating men on the importance of good health seeking behavior will translate into good health seeking behavior for their female dependents as well. A study on women's decisions to access maternal healthcare in Ghana concluded that interventions should target husbands as, in almost 50% of cases where services were not accessed, husbands made that decision [19]. The role of men in health decision-making in the Ghanaian society cannot therefore be underestimated and ensuring that adolescent males are well educated on breast cancer can result in positive influences on their future wives and other female relations. Additionally, while most breast cancer initiatives have targeted adult females, targeting adolescents has been suggested as a means for developing good health seeking habits which can be passed on to family and friends as well as subsequent generations [12, 17, 20, 21]. Educating the younger generation about breast cancer and increasing their awareness should produce subsequent generations who are knowledgeable about breast cancer and thus more likely to detect breast cancer earlier and experience better health outcomes [3, 12, 22].

Incorporating breast cancer education into schools is an effective way of increasing knowledge of breast cancer widely among adolescent females and males [8, 10, 17, 21, 23]. Hence, this study sought to determine the baseline levels of knowledge of breast cancer in female and male adolescents in senior high schools (SHSs). This will inform the implementation of a previously piloted breast cancer educational intervention which utilized a multitool approach to education using drama, a PowerPoint presentation, question and answer session, and educational leaflets in two SHSs in Ghana [24].

## 2. Methodology

This multisite, cross-sectional study was conducted to assess the baseline knowledge of breast cancer in SHS students in the Greater Accra and Central regions of Ghana. Ghana is divided into 16 regions with the Greater Accra and Central regions being the first and fourth most populous regions. The Greater Accra region is known to be the most economically developed region, and the Central region is the poorest region in southern Ghana. The Greater Accra region has 46 schools and the Central region 75 schools. Together these two regions house 17% of the 704 SHSs in the country.

SHSs offer a 3-year educational program and are either single sex or coeducational schools. Seven (7) schools from each region were selected by cluster sampling. The schools were approached through partnership with the Ghana Education Service who approved the study to be carried out in their schools and contacted each school directly about study participation. The study procedure was explained and written informed consent taken from heads of participating schools who serve as guardians of the students in the SHS as their parents are not available in the boarding schools.

Self-administered paper questionnaires covering knowledge of breast cancer and BSE practice were distributed to all second year SHS students at a single point in time. The purpose of the study was explained and informed written assent obtained from all who were willing to participate voluntarily. The questions consisted of 23 true/false and multiple-choice questions which covered four domains regarding breast cancer: general knowledge (4), symptoms (7), risk factors (7), and BSE/breast cancer screening (5). The boys were exempted from answering the two questions on the practice of BSE.

### 2.1. Statistical Analysis

Each correct answer was assigned one (1) point, and each incorrect answer was assigned a score of zero (0). If a question was left blank or answered “do not know,” this also was assigned a score of zero (0). Each of the questions in the four domains was assigned equal weight/value. The percentage of correct answers given by the students on each question was then calculated. For the four domains, the score was calculated by summing the score of all the questions in that domain ranging from 0 to 4 for the breast cancer general knowledge domain, 0–7 for the symptoms of breast cancer, 0–5 for BSE/breast screening domains, and 0–7 for the risk factors of breast cancer domain. The percentage scores were then calculated. Total score for each domain was categorized as either adequate knowledge (≥ 50% of questions answered correctly) or inadequate knowledge (< 50% of questions answered correctly). The overall score was calculated by summing all the scores for the four domains. Logistic regression was used to determine factors (school type, gender, and region) associated with each of the knowledge domains. Continuous variables were summarized as means and standard deviations (SDs) and categorical variables as count and percentages. Stata 14.0 was used for the statistical analysis, and a *p* value less than 0.05 was considered statistically significant.

### 2.2. Ethical Considerations

The Institutional Review Board of Korle Bu Teaching Hospital for Medical Research (KBTH-IRB) (study protocol ID KBTHIRB/00063/2018) granted ethical approval. Heads of all the participating schools approached by the Ghana Education Service gave written informed consent. Additionally, each student study participant gave written informed consent after the procedure had been explained and the option to withdraw at any stage was made clear.

## 3. Results

A total of 9767 students from 14 schools participated in the study. Ten of these schools were coeducational while the remaining four were girls-only schools. Participants from the coeducational schools formed 74% (7236) of the total number of surveyed students, while 26% (2531) of participants were from the girls-only schools. While there were 7 of the 14 schools represented in each region, 61% of the total number of participants were from schools in the Central region. Of all participants from all schools, 68% were female, 23% were male, and 9% did not identify their gender. The mean age of the students was 16.9 ± 1.2 years.

### 3.1. General Knowledge of Breast Cancer

Regarding the four general knowledge questions posed to the students about breast cancer, 75% knew that breast cancer can occur in young women less than 30 years of age. Approximately 65% of the students knew that breast cancer can occur in men and pregnant women and that breast cancer can be a curable disease. However, between 15% and 28% of participants did not know the answers to the questions posed on general knowledge of breast cancer (Table 1).

### 3.2. Knowledge of Breast Cancer Symptoms

On knowledge of breast cancer symptoms, 81% and 73% of students, respectively, correctly identified a breast “lump” or mass and breast swelling as potential symptoms of breast cancer. Approximately 60% of students correctly identified a sore or rash on the breast and fluid nipple discharge as concerning symptoms of breast cancer. However, only about a third of high school students recognized a change in the direction of the nipple, such as inversion, as concerning, and only 17% recognized an axillary “lump” or mass as a potential symptom of a breast cancer diagnosis. Most students (68.5%) incorrectly believed breast cancer to start with pain in the affected breast. Other than breast “lumps,” it is important to note that 20%–40% of students responded that they did not know if these symptoms were associated symptoms of breast cancer or not (Table 1).

### 3.3. Knowledge of Breast Cancer Risk Factors

Knowledge of risk factors of breast cancer was generally low with less than half of the participants knowing about family history of breast cancer, excessive alcohol intake, and lack of exercise as factors that increase a woman's risk of developing breast cancer and of breastfeeding reducing a woman's risk. However, multiparity as a factor that reduces the risk of breast cancer was correctly identified by 69% of students. Concerning the widely held misconceptions about risk factors, less than half of the students knew that oral stimulation of the breast by men is not a risk factor and only 6% knew that keeping a handkerchief or mobile phone in brassieres was not a risk factor (Table 2).

### 3.4. Knowledge of BSE and Breast Cancer Screening

Although 78% of students had heard about BSE, only 43% had ever practiced it and fewer still (19%) had done so in the last 3 months (not shown in table).

Table 2 shows that with breast screening and BSE, only 4 in 10 students knew the best time to carry out a BSE and also the recommended frequency of doing so. Almost 50% of the students either did not know the age at which the first screening mammogram is recommended or answered incorrectly with only 3% correctly identifying the recommended age.

### 3.5. Overall Knowledge of Breast Cancer

While greater than 80% of students demonstrated adequate general knowledge of breast cancer, only 54%, 34%, and 21% of students demonstrated adequate knowledge of breast cancer symptoms, risk factors, and early detection practices, respectively. The overall level of knowledge of breast cancer among the high school students was deemed inadequate in more than half of the high school students participating in this study (Table 3).

### 3.6. Association Between Demographics and Levels of Knowledge

Table 4 shows the sociodemographic factors associated with the different knowledge domains. In the multivariable analysis, female students showed statistically significant adequate levels of knowledge in all domains of breast cancer knowledge assessed (*p* < 0.05) compared with the male students (Table 4). It is thus not surprising that students at the girls-only schools demonstrated statistically significant adequate knowledge regarding general knowledge and symptoms of breast cancer compared with students at coeducational schools (*p* < 0.05) (Table 4). Students from schools located in the Central region had statistically significant adequate levels of general knowledge of breast cancer, breast cancer symptoms, and BSE/breast cancer screening compared to students from schools in the Greater Accra region (*p* < 0.001) (Table 4).

## 4. Discussion

This study assessed the knowledge of breast cancer among adolescents in SHSs in the southern part of Ghana. The overall knowledge of breast cancer was found to be inadequate. Knowledge of risk factors, BSE, and breast screening was particularly inadequate. Boys were found to have a statistically significantly inadequate knowledge compared to girls and Central region schools and girls-only schools also had statistically significantly adequate knowledge in most of the domains.

In a LMIC country such as Ghana with resource limitations and high burden of late presentation with late stage disease and growing projected incidence rates in the years ahead, creation of a national breast cancer screening program is not feasible as a short-term goal [3]. However, efforts can be made to improve patient outcomes with the first pillar of the WHO's Global Breast Cancer Initiative: health promotion for early detection [3]. This goal must be achieved through increasing breast health awareness and breast cancer education. Being knowledgeable about breast cancer, its features, and risk factors and being able to correctly perform a BSE are especially important where routine mammographic screening is not available, as > 90% of breast cancers in such individuals are diagnosed by self-detection [3]. Thus, it is essential for LMICs including Ghana to assess baseline knowledge and practices relating to breast cancer. There is little data available on this in Ghana among adolescents. Results from this study can be used to formulate an educational package that will address the gaps that are detected. This can be integrated into the country's school heath curriculum to have a national reach and impact.

This study found that general knowledge about breast cancer was high with 84% of the answers being correct. Specifically, about 65% of the students knew that breast cancer can be cured and can affect men as well as pregnant women and 75% of them knew that young women could be affected by breast cancer. But as many as 25%–35% of the students were not aware of these facts abouts breast cancer with a third of them not knowing that breast cancer can be cured. This lack of knowledge can influence their choices when faced with breast cancer. High levels of knowledge have been reported among American university students in a State Comprehensive University in South Carolina, where 93% of the students, majority of whom were younger than 20 years, had satisfactory or good knowledge of breast cancer [17]. This shows that it is possible to have higher levels of knowledge among students and should be our target.

Similar levels of fund of knowledge have been reported in other studies on breast cancer. In this study, 54% of the participants had adequate knowledge of the breast cancer symptoms, similar to that found in other studies across different income strata (43%–59%) [16, 21, 25]. A breast “lump” is a commonly known symptom of breast cancer [16, 23] and not surprisingly majority of the students in this study correctly identified a breast “lump” as a feature of breast cancer. However, knowledge of other symptoms of breast cancer such as a rash or change in direction of the nipple, nipple discharge, and axillary swelling was not as high as also reported by other studies [11, 16, 22, 23]. It is therefore important that educational activities emphasize the nonlump symptoms of breast cancer. In particular, 87% of the students in this study either erroneously thought that or did not know whether or not breast cancer starts with pain in the breast. Similarly, in an Iranian study among high school girls, 37% associated painful breast lumps with breast cancer [21]. This is a very common misconception which leads to women not reporting early to health facilities when they first notice painless breast lumps, as they do not associate them with breast cancer. The fact that breast cancer is usually painless when it starts should be stressed in educational campaigns to help dispel this misconception.

This study showed inadequate knowledge about risk factors for breast cancer with only a third of the participants having adequate knowledge. This is not different from what other studies from across countries of different income strata have found with poor knowledge of breast cancer risk factors [11, 17, 20, 21, 25]. Misconceptions such as breast cancer being caused by putting objects such as mobile phones or money into brassieres were only identified by 7% of our study participants as false. Such misconceptions may not be unique to Ghana as some American university students thought wearing tight brassieres (11%) and trauma to the breast (15%) could result in breast cancer [17]. Also of concern is the fact that just about a third of our study participants recognized that lack of exercise and excessive alcohol are risk factors for breast cancer. These are modifiable risk factors that can be avoided to reduce breast cancer risk. Awareness of such risks encourages adoption of preventive measures. A study in Nigeria also found that majority of participants were not aware of these lifestyle factors including obesity being associated with increased risk of breast cancer [11]. This again calls for emphasis on the risk factors of breast cancer especially the modifiable risks during educational activities to raise awareness and positively influence health habits [3, 12].

Regarding breast screening, this study found that only 3% of the participants correctly identified the recommended age for starting mammograms. This is not unusual given that there is no national mammographic screening program in Ghana. However, the authors do feel that this information is important to assess and teach. Another study in Iran also showed a low level of knowledge as to when to start mammograms (15%) [21]. Similarly, the study in Iran identified that > 87% of the students were unaware or had inadequate knowledge about breast cancer screening [21] while in another study in Sri Lanka about a third of the participants did not recognize mammography as an effective screening tool [23]. Although there is no national screening program in Ghana, having adequate knowledge about when to start screening with mammograms is necessary as opportunistic screening is available and individuals with the requisite knowledge can take advantage of that.

Regarding BSE, 78% of the participants in this study had heard about it but < 50% had ever done it with an even smaller percentage of 19% who had done so in the last 3 months. Overall assessment of knowledge and practice of BSE was adequate in just 21% of the participants. Considering that the mean age of the students was 16 years and considering that it is very unusual for adolescent females to develop breast cancer, it is not surprising that knowledge and practice of BSE were rather poor. Studies done among older individuals showed increased awareness and practice of BSE, but the proportions were still inadequate [8]. A study in Nigeria to improve knowledge on breast cancer and BSE found that 68% of the students had heard about BSE but only 4% practiced it. Interestingly, that study also identified that not all the students who had heard about BSE knew that it could help identify breast cancer [11]. Several studies also assessing knowledge and practice of BSE found this to be inadequate as majority of participants did not know the age to begin BSE, how to perform it, the frequency of doing it, and the best time to do it [8, 20–23, 25]. This emphasizes the need to explain fully what BSE is and hopes to achieve, how it should be performed, when, and how often. A study conducted in Ghana found that for patients who performed BSE, they did so primarily because they appreciated its benefit to them [8] while another study showed that those who did not practice it were not familiar with how to do so [20]. In a study done in Ibadan, an association was found between knowledge of BSE and its practice with those who had good knowledge being more likely to have positive attitudes toward BSE [22]. BSE has been found to be a valuable tool in awareness education in LMICs, and its practice should be encouraged with the aim of detecting early warning signs of breast cancer which should lead the individual to seek the appropriate healthcare [3, 14]. Educating adolescents on BSE is aimed at equipping them with the requisite knowledge needed prior to the recommended age of 20 years to start practicing BSE [12]. The national strategy for cancer control in Ghana recommends that all girls be taught BSE at the age of 16 years [26] and BSE can be taught earlier in order to better achieve this aim.

Overall, the female students had a statistically significant adequate level of knowledge in all domains of breast cancer knowledge assessed compared to the males. This was also found in other studies where the females were more likely to have better breast cancer knowledge compared to the males [17, 27]. This is most likely explained by the fact that breast cancer affects mostly women [1] and hence girls are more likely to be interested in finding out more about it. Secondly, health talks on breast cancer usually target females so they may have previously had more opportunities to be educated on breast cancer. It is therefore not surprising that in the girls-only schools, there was statistically significant adequate knowledge compared to the mixed schools. This pertained in all the domains assessed except for BSE and screening which was generally poor across all school types but still significantly better among females. More efforts should therefore be made to include males in breast cancer awareness and educational activities [12]. In certain communities in Ghana, culturally women depend on their male partner's consent and/or financial support to access healthcare, and this is known to contribute to delays in breast cancer diagnosis. This makes education of males crucial. This study was not able to explain the statistically significant adequate knowledge levels of breast cancer in the Central region schools compared to the Greater Accra region. All the schools in this study are boarding facilities and, as is the case in Ghana, students are enrolled from across the 16 regions of the country and study findings might not be attributed to the geographic location of the school.

The strength of this study lies in the large sample size. The authors believe the findings of this study are generalizable to at least the southern sector of Ghana and most likely a reflection of breast cancer knowledge in SHS students across the country. A limitation of this study includes a possible recall bias as participants can guess the answer with a close-ended questionnaire. The cross-sectional design also does not allow changes in knowledge to be assessed overtime but the next study to be carried out by the authors will be designed to test knowledge over time. In addition, the questionnaire used in this study was adapted from a previous pilot study, where it was assessed for face and content validity by experts in the field. However, no formal psychometric evaluation of its reliability was conducted or reported. This represents a limitation, as the absence of reliability metrics may affect the interpretability and generalizability of the findings. Future studies are encouraged to perform comprehensive validation procedures, including assessments of construct validity and internal consistency reliability. The overall knowledge score combined multiple domains that contained unequal numbers of questions. This may have introduced bias by disproportionately weighting domains with more items, thereby skewing the representation of participants' overall knowledge. Future studies should consider standardizing the number of questions per domain or using domain-specific scores to provide a more balanced assessment.

## 5. Conclusion

The overall knowledge of breast cancer among SHS students in two regions of southern Ghana was found to be inadequate specifically regarding knowledge about breast cancer risk factors, BSE, and breast screening. Although adequate knowledge was found in some aspects of the general knowledge and symptoms of breast cancer, many students thought that breast cancer starts with pain and were unaware of symptoms concerning for a breast cancer diagnosis other than a breast mass. The majority of students were not aware of lifestyle-related risk factors, the recommended age to start breast screening with mammography, and how to perform an appropriate BSE. Male students, students from the Greater Accra region, and students in coeducational schools had scores deemed inadequate more often than the female high school students, students attending girls-only schools, or students from the Central region; this reached statistical significance. Given these results, there is the urgent need to step up breast cancer educational activities in SHSs across the country, with emphasis on the behavioral risk factors, nonlump (mass) symptoms, the painless nature of initial cancerous breast lumps, BSE, and breast screening. Coeducational schools and boys should be given equal attention as girls and girls-only schools. Finally, it is important to acknowledge that 53% of the students did not have a level of knowledge deemed to be adequate. Such knowledge gaps can influence their choices if faced with a breast cancer. Hence, the goal should be to increase the proportions of students with accurate knowledge about breast cancer to as close to 100% as possible.

Results from this study can be used to formulate an educational plan that will address the gaps that are detected. This can be integrated into the country's school heath curriculum to have a national reach and impact. Adolescent males and gender-inclusive messaging should be included in breast cancer education. Trained school nurses and teachers are well positioned to deliver these educational interventions which will ensure sustainability over the years.

## Figures and Tables

**Table 1:** General knowledge and symptoms of breast cancer.

**Domain**	**Characteristic**	**Correct answer**	**Incorrect answer**	**Do not know**
		**n** **, %** ^**a**^	**n** **, %** ^**a**^	**n** **, %** ^**a**^
*General breast cancer knowledge*	Breast cancer is curable	6239 (63.9)	1583 (16.2)	1945 (19.9)
	Men can have breast cancer	6246 (64.0)	1886 (19.3)	1635 (16.7)
	Women < 30 years can have breast cancer	7338 (75.1)	879 (9.0)	1550 (15.9)
	Pregnant women can get breast cancer	6486 (66.4)	602 (6.2)	2679 (27.4)

*Knowledge of breast cancer symptoms*	Lump in the breast	7903 (80.9)	461 (4.7)	1403 (14.4)
	Swelling of breast	7077 (72.5)	629 (6.4)	2061 (21.1)
	Lump in the armpit	1730 (17.7)	5130 (52.5)	2907 (29.8)
	Sore/rash on the breast	5870 (60.1)	1223 (12.5)	2674 (27.4)
	Change in direction of the nipple	3283 (33.6)	2738 (28.0)	3746 (38.4)
	Fluid discharge from the nipple	6437 (65.9)	847 (8.7)	2483 (25.4)
	Usually starts with pain in the breast	1228 (12.6)	6689 (68.5)	1850 (18.9)

^a^Row percentages.

**Table 2:** Knowledge of breast cancer risk factors and knowledge of BSE and screening.

**Domain**	**Characteristic**	**Correct answer**	**Incorrect answer**	**Do not know**
		**n** **, %** ^**a**^	**n** **, %** ^**a**^	**n** **, %** ^**a**^
*Knowledge of breast cancer risk factors*	Family member had breast cancer	4199 (43.0)	3192 (32.7)	2376 (24.3)
	Breastfeeding	4833 (49.5)	2464 (25.2)	2470 (25.3)
	Having more than 5 children	6735 (69.0)	521 (5.3)	2511 (25.7)
	Excessive alcohol intake	3315 (33.9)	2771 (28.4)	3681 (37.7)
	Lack of exercise	3191 (32.7)	3366 (34.5)	3210 (32.9)
	Keeping objects in brassiere	633 (6.5)	8111 (83.0)	1023 (10.5)
	Men sucking breast	4536 (46.4)	1902 (19.5)	3329 (34.1)

*Knowledge of BSE and screening*	Frequency of doing BSE	4082 (41.8)	3641 (37.3)	2044 (20.9)
	Best time to do breast self-examination	4001 (41.0)	5000 (51.2)	766 (7.8)
	Age a woman should have first mammogram	318 (3.3)	4769 (48.8)	4680 (47.9)

^a^Row percentages.

Abbreviation: BSE, breast self-examination.

**Table 3:** Domains of breast cancer knowledge analysis.

**Characteristic**	**Adequate**	**Inadequate**
	**n** **, %** ^**a**^	**n** **, %** ^a^
General knowledge of breast cancer	8217 (84.1)	1550 (15.9)
Knowledge of breast cancer symptoms	5282 (54.1)	4485 (45.9)
Knowledge of breast cancer risk factors	3354 (34.3)	6413 (65.7)
Knowledge of BSE and screening	2083 (21.3)	7684 (78.7)
Overall knowledge	4564 (46.7)	5203 (53.3)

^a^Row percentages.

Abbreviation: BSE, breast self-examination.

**Table 4:** Factors associated with adequacy of knowledge of breast cancer.

	**General knowledge on BC**		**Crude odds ratio [95% CI]**	**p** ** value**	**Adjusted odds ratio [95% CI]**	**p** ** value**
	**Adequate**	**Inadequate**				
	**n** **, %**	**n** **, %**				

School type						
Coeducational	5944 (82.1)	1292 (17.9)	1.00		1.00	
Girls-only	2273 (89.8)	258 (10.2)	1.91 [1.66–2.21]	< 0.001	1.53 [1.31–1.79]	***< 0.001***
Sex						
Female	5771 (87.0)	859 (13.0)	1.00		1.00	
Male	2446 (78.0)	691 (22.0)	0.53 [0.47–0.59]	< 0.001	0.61 [0.54–0.69]	***< 0.001***
Region						
Greater Accra	3131 (82.0)	686 (18.0)	1.00		1.00	
Central	5086 (85.5)	864 (14.5)	1.29 [1.16–1.44]	< 0.001	1.29 [1.16–1.44]	***< 0.001***

	**Knowledge of BC symptoms**		**Crude odds ratio [95% CI]**	**p** ** value**	**Adjusted odds ratio [95% CI]**	**p** ** value**
	**Adequate**	**Inadequate**				
	**n** **, %**	**n** **, %**				

School type						
Coeducational	3773 (52.1)	3463 (47.1)	1.00		1.00	
Girls-only	1509 (59.6)	1022 (40.4)	1.36 [1.24–1.46]	< 0.001	1.18 [1.06–1.30]	***0.002***
Sex						
Female	3799 (57.3)	2831 (42.7)	1.00		1.00	
Male	1483 (47.3)	1654 (52.7)	0.67 [0.61–0.73]	< 0.001	0.71 [0.65–0.78]	***< 0.001***
Region						
Greater Accra	1975 (51.7)	1842 (48.3)	1.00		1.00	
Central	3307 (55.6)	2643 (44.4)	1.17 [1.08–1.27]	< 0.001	1.17 [1.07–1.27]	***< 0.001***

	**Knowledge of BC risk factors**		**Crude odds ratio [95% CI]**	**p** ** value**	**Adjusted odds ratio [95% CI]**	**p** ** value**
	**Adequate**	**Inadequate**				
	**n** **, %**	**n** **, %**				

School type						
Coeducational	2427 (33.5)	4809 (66.5)	1.00		1.00	
Girls-only	927 (36.6)	1604 (63.4)	1.15 [1.04–1.26]	0.005	1.07 [0.97–1.19]	0.186
Sex						
Female	2365 (35.7)	4265 (64.3)	1.00		1.00	
Male	718 (31.5)	1562 (68.5)	0.83 [0.76–0.91]	< 0.001	0.85 [0.77–0.94]	***0.002***
Region						
Greater Accra	1282 (33.6)	2535 (66.4)	1.00		1.00	
Central	2072 (34.8)	3878 (65.2)	1.06 [0.97–1.15]	0.209	1.06 [0.97–1.15]	0.219

	**Knowledge of BSE and screening**		**Crude odds ratio [95% CI]**	**p** ** value**	**Adjusted odds ratio [95% CI]**	**p** ** value**
	**Adequate**	**Inadequate**				
	**n** **, %**	**n** **, %**				

School type						
Coeducational	1514 (20.9)	5722 (79.1)	1.00		1.00	
Girls-only	569 (22.5)	1962 (77.5)	1.10 [0.98–1.22]	0.100	0.96 [0.85–1.08]	0.499
Sex						
Female	1530 (23.1)	5100 (76.9)	1.00		1.00	
Male	372 (16.3)	1907 (83.7)	0.71 [0.64–0.79]	< 0.001	0.71 [0.63–0.79]	***< 0.001***
Region						
Greater Accra	699 (18.3)	3118 (81. 7)	1.00		1.00	
Central	1384 (23.3)	4566 (76.7)	1.35 [1.22–1.50]	< 0.001	1.34 [1.21–1.49]	***< 0.001***

	**Overall knowledge**		**Crude odds ratio [95% CI]**	**p** ** value**	**Adjusted odds ratio [95% CI]**	**p** ** value**
	**Adequate**	**Inadequate**				
	**n** **, %**	**n** **, %**				

School type						
Coeducational	3255 (45.0)	3981 (55.0)	1.00		1.00	
Girls-only	1309 (51.7)	1222 (48.3)	1.31 [1.20–1.43]	< 0.001	1.13 [1.02–1.25]	***0.018***
Sex						
Female	3318 (50.1)	3312 (49.9)	1.00		1.00	
Male	891 (39.1)	1389 (60.9)	0.66 [0.60–0.72]	< 0.001	0.69 [0.63–0.76]	***< 0.001***
Region						
Greater Accra	1649 (43.2)	2168 (56.8)	1.00		1.00	
Central	2915 (49.0)	3035 (51.0)	1.26 [1.16–1.37]	< 0.001	1.26 [1.16–1.37]	***< 0.001***

*Note:* Significant *p* values in bold.

## Data Availability

The data and materials for this study are freely available at 10.6084/m9.figshare.27704829.v1.
